# Clinical Outcome and Quality of Life of Multimodal Treatment of Extracranial Arteriovenous Malformations: The APOLLON Study Protocol

**DOI:** 10.1007/s00270-022-03296-8

**Published:** 2022-10-19

**Authors:** Vanessa F. Schmidt, Max Masthoff, Veronika Vielsmeier, Caroline T. Seebauer, Özlem Cangir, Lutz Meyer, Antje Mükke, Werner Lang, Axel Schmid, Peter B. Sporns, Richard Brill, Walter A. Wohlgemuth, Natascha Platz Batista da Silva, Max Seidensticker, Regina Schinner, Julia Küppers, Beate Häberle, Frank Haubner, Jens Ricke, Martin Zenker, Melanie A. Kimm, Moritz Wildgruber, for the APOLLON investigators

**Affiliations:** 1 Department of Radiology, University Hospital, LMU Munich, Munich, Germany; 2 Clinic for Radiology, University Hospital Muenster, Muenster, Germany; 3 Department of Otorhinolaryngology, University Hospital Regensburg, Regensburg, Germany; 4 Department of Pediatric Surgery, Center for Vascular Malformations, Klinikum Barnim GmbH, Werner Forssmann Hospital, Eberswalde, Germany; 5 Department of Vascular Surgery, University Hospital Erlangen, Erlangen, Germany; 6 Department for Radiology, University Hospital Erlangen, Erlangen, Germany; 7 Department of Neuroradiology, Clinic of Radiology and Nuclear Medicine, University Hospital Basel, Basel, Switzerland; 8 Clinic and Policlinic of Diagnostic Radiology, Martin-Luther University Halle-Wittenberg, Halle (Saale), Germany; 9 Department of Radiology, University Hospital Regensburg, Regensburg, Germany; 10 Department for Pediatric Surgery, University Hospital, LMU Munich, Munich, Germany; 11 Department of Otorhinolaryngology, University Hospital, LMU Munich, Munich, Germany; 12 Institute for Human Genetics, Otto von Guericke University of Magdeburg, Magdeburg, Germany

**Keywords:** APOLLON trial, AVM, Multimodal therapy, Vascular malformations, QoL

## Abstract

**Purpose:**

Arteriovenous malformations (AVMs) as rare diseases are diagnostically and therapeutically challenging. Due to the limited evidence regarding treatment outcome, prospective data are needed on how different treatment regimens affect outcome. The aims of this prospective trial are to determine effectiveness, safety, and clinical outcome of multimodal treatment in patients with extracranial AVMs.

**Materials and Methods:**

After clinical and magnetic resonance imaging (MRI)-based diagnosis and informed consent, 146 patients (> 4 years and < 70 years) undergoing multimodal therapy in tertiary care vascular anomalies centers will be included in this prospective observational trial. Treatment options include conservative management, medical therapy, minimally invasive image-guided procedures (embolization, sclerotherapy) and surgery as well as combinations of the latter. The primary outcome is the patient-reported QoL 6 months after completion of treatment using the short form-36 health survey version 2 (SF-36v2) and the corresponding short form-10 health survey (SF-10) for children. In addition, clinical presentation (physician-reported signs), MRI imaging (radiological assessment of devascularization), recurrence rate, and therapeutic safety will be analyzed. Further follow-up will be performed after 12, 24, and 36 months. Moreover, liquid biopsies are being obtained from peripheral blood at multiple time points to investigate potential biomarkers for therapy response and disease progression.

**Discussion:**

The APOLLON trial is a prospective, multicenter, observational open-label trial with unequal study groups to generate prospective evidence for multimodal treatment of AVMs. A multicenter design with the potential to assess larger populations will provide an increased understanding of multimodal therapy outcome in this orphan disease.

**Trial Registration:**

German Clinical Trials Register (identification number: DRKS00021019) https://www.drks.de/drks_web/navigate.do?navigationId=trial.HTML&TRIAL_ID=DRKS00021019.

**Supplementary Information:**

The online version contains supplementary material available at 10.1007/s00270-022-03296-8.

## Introduction

Vascular malformations, based on an angiogenic and vasculogenic disorder, are ‘orphan’ and complex diseases being present already at birth (even if asymptomatic) and never regress spontaneously. They may be quiescent for a long time before mechanical stimuli (like incomplete interventions) or hormonal influence (characteristically around puberty or during pregnancy) can stimulate them to gain volume and size and altered hemodynamics [1]. The classification of vascular malformations (simple, combined, of major named vessels, associated with other anomalies) according to the International Society for the Study of Vascular Anomalies (ISSVA) [2–4] is crucial both for treatment decision and prognosis [5]. In general, arteriovenous malformations (AVMs) represent the minority (< 10%) of vascular malformations [6, 7]. They result from an abnormal connection between arteries and veins without a capillary bed in between [8–10], continuously expanding over time. Due to the fast-flow shunting, hemodynamic effects such as local hypervascularity, increased venous pressure, and steal phenomenon occur, which can result in peripheral ischemia distal to the malformation [1, 11, 12]. Thus, the patients may present with local pain, bleeding, hyperemia, ulceration, and gangrene up to high-output cardiac failure [13, 14]. In some cases, tissue overgrowth may accompany the affected region. Since AVMs demonstrate trans-spacial tissue penetration and have high surgical recurrence rates, primary surgical resection has become less common. The value of surgery mostly lies in resection of previously embolized lesions that are only residually perfused, to manage complications following embolization as well as to reconstruct larger defects occurring after embolization [15]. Once the decision for invasive treatment is made, complete embolization of the AVM is the goal. Incomplete embolization can result in a rapid increase of the lesion [16]. In cerebral AVMs, partial embolization results in a release of various angiogenic growth factors from the AVM which promotes progression of growth rather than regression [17, 18]. However, there is a debate if cerebral AVMs behave similarly to peripheral AVMs; however, mere extrapolation of knowledge on pathophysiological and molecular aspects may not be appropriate [19]. Besides embolization and surgical resections, conservative treatment options including compression garments may serve as supplementary or supportive. Pharmaceutical treatments, mainly as off-label use from oncology targeting the effects of characteristic mutations such as phosphatidylinositol-4,5-bisphosphate 3-kinase catalytic subunit alpha (PIK3CA) or rat sarcoma (RAS)/mitogen-activated protein kinase (MAPK), are more frequently used for targeted treatment in AVM [20, 21].

While AVMs present diagnostic and therapeutic challenges affecting mainly children and young adults [22], to date, no reliable data from national and international vascular anomalies’ centers exist. The majority of available data exists in form of case reports and retrospective studies with limited evidence regarding the outcome of treatment for AVMs [23–28]—therefore, prospective data are needed.

## Study Design and Registration

The APOLLON trial is a prospective, multicenter, observational, and open-label study with and additional biobanking. It is a collaboration between German, Austrian, and Swiss vascular anomalies centers and registered with the German Clinical Trials Register (DRKS, identification number: DRKS00021019). All study procedures are in accordance with the 1964 Helsinki Declaration and its later amendments. Ethical approval by the local Ethics Committee (University Hospital, LMU Munich) has been granted, Project No: 20-445 (09/03/2020). The study protocol can be found as Supplementary File 1.

## Study Objective

The study’s objective is to determine the effectiveness, safety, and clinical outcome including health related QoL of multimodal treatment of AVMs. Treatment options include conservative management, medical therapy, minimally invasive image-guided procedures (embolization, sclerotherapy), and surgery as well as combinations of the above. Additionally, facultative liquid biopsies are being collected from peripheral blood to investigate potential biomarkers (cytokines, chemokines, growth factors) and circulating endothelial cells (CEC) as markers for therapy response or disease progression [29, 30].

## Inclusion and Exclusion Criteria

Adults (< 70 years) and children (> 4 years) with extracranial AVMs are similarly included. Full inclusion and exclusion criteria are provided in Table 1.

**Table 1:** Full inclusion and exclusion criteria

Inclusion criteria^a^	Exclusion criteria
Age: > 4 years, < 70 years	AVMs located in the central nervous system
Simple peripheral (= extracranial) AVMs according to the ISSVA classification	AVMs located in abdominal parenchymal organs or the gastrointestinal tract
Combined^b^ vascular malformations and AVMs associated with other anomalies (e. g. Parkes Weber, PTEN hamartoma, HHT) according to the ISSVA classification	Other high-flow vascular anomalies (e. g. vascular tumors)
Patients with first line therapy or patients with previous alternative therapies in whom the previous treatments did not lead to durable symptom improvement in case of a therapy-free interval of 12 months	Concomitant life-limiting diseases (such as cancer)
Absence of any psychological, familial, sociological, or geographical condition with potential negative impact	Acute inflammatory diseases or acute bacterial superinfection of an AVM related ulceration
Written informed consent	Patients with contraindications for invasive treatments
	Contrast agent intolerance or renal insufficiency (GFR < 30 ml/min)
	Impaired coagulation status (Platelet count < 50.000/μl, aPTT > 50 s, INR > 1.5
	ECOG performance > 1
	Pregnant or breast-feeding women
	Inability of the patient/parents to understand or follow the study protocol e. g. due to impaired mental health status
	Inability to access the AVM lesion due to anatomical or pathoanatomical reason

*aPPT* activated partial thromboplastin time, *AVMs* arteriovenous malformations, *ECOG* Eastern Cooperative Oncology Group, *GFR* glomerular filtration rate, *HHT* hereditary hemorrhagic telangiectasia, *INR* international normalized ratio, *ISSVA* international society for the study of vascular anomalies, *PTEN* phosphatase and tensin homolog

^a^Each patient and the corresponding diagnosis is discussed in an interdisciplinary board for vascular anomalies before initiation of treatment

^b^Defined as two or more vascular malformations found in one lesion

## Study Cohort

A total of 146 patients with simple or combined extracranial AVMs (except AVMs located in abdominal parenchymal organs or the gastrointestinal tract) will be included across the participating centers.

## Description of Procedures

### Screening

Potential study participants are screened on an interdisciplinary basis according to the inclusion criteria through the participating vascular anomalies’ centers. The screening performed within the routine clinical workup includes laboratory analysis and pre-interventional ultrasound and magnetic resonance imaging (MRI) to confirm the diagnosis and to identify possible treatment options. Digital subtraction angiography (DSA) will not be routinely applied as a mere diagnostic modality before study inclusion; however, DSA being performed before minimally invasive treatment will be analyzed to confirm both the presence and the subtype of AVM according to the Cho classification [8]. Furthermore, the maximum size of the initial AVM (volumetric measurements) will be assessed based on the cross-sectional imaging data. After detailed study information and informed consent of the patients (and the parents in case of pediatric patients), the patient is included in the study. The follow-up assessment is composed of the same subjective (SF-36v2/SF-10, visual analogue scale of pain) and objective (clinical presentation, AVM imaging) measures as compared to baseline; detailed description is provided below (outcome measures). We have defined the indication for treatment according to the following pathway: indication for any kind of treatment is a symptomatic AVM. Treatment decisions are therefore guided following the Schobinger classification. Stage I is usually treated conservatively. Stage II-IV is an indication for invasive treatment. For most indications, image-guided embolization is the first treatment modality of choice, surgery is either applied in a reconstructive manner when needed for repair of associated tissue damage or in case of complete/near-complete embolization resection can be performed in order to reduce recurrence rates. Regarding small AVMs which can be entirely resected (comparable to an R0 tumor resection), definite surgery without prior embolization may be performed. As stated above exception from this approach especially apply to certain AVM localizations, for further information, see Table 2. Conservative management and/or invasive treatment is initiated following interdisciplinary board consensus in a specialized vascular anomalies center. With regard to minimal requirements, each participating vascular anomalies center has to provide at least expertise in the following subspecialties: interventional radiology (specialized in vascular anomalies procedures), plastic and reconstructive surgery, pediatric surgery, pediatrics with expertise in targeted therapies (most frequently within pediatric hematology and oncology departments), otorhinolaryngology/head and neck surgery, as well as orthopedic surgery with expertise in soft tissue surgery. All centers contributing are collaborating between each other both via online case conference compared to interdisciplinary tumor boards. Additionally, online study meetings are performed on a monthly basis.

**Table 2:** Schematic overview of treatment indications

Asymptomatic AVM^a^ Schobinger I	Symptomatic AVM Schobinger II–IV
Conservative treatment	Invasive treatment
Compression garments (if possible, prophylactic)	Image-guided embolotherapy (in most cases first treatment of choice)
	Additional surgery (for tissue repair and to reduce risk of recurrence)
	Primary surgery (for definite resection of small AVMs)
	Further (for symptom improvement): Compression garments (if possible), medical treatment, physiotherapy

*AVM* arteriovenous malformation

^a^Exceptional (asymptomatic lesions with indication for treatment): AVMs of the face in case of functional/aesthetic impairment, AVMs of distal extremities with risk of ischemia due to shunting

### Multimodal Treatment

As the study is conducted as an observational trial, all treatment procedures are considered part of the clinical routine. Due to the multimodal treatment in everyday practice, certain comparisons of the most frequent treatment approaches are anticipated at the end. Therapy options being amenable for inclusion include image-guided minimally invasive intervention (embolization), surgery (resection and if necessary plastic reconstruction), medical therapy and conservative treatment including compression therapy. Venous hypertension, insufficiency, and ulcer may be the results of a progredient/decompensated symptomatic AVM caused by AV shunting, increased vein perfusion, and consequent vessel wall weakness. Yet, also in compensated clinical stages, compression can reduce the volume of the venous component of the AVM and alter hemodynamics, thereby potentially reducing pain, hyperhidrosis, and the feeling of heaviness and pressure, especially in the lower extremities [31].

Targeted medical therapy can either be applied alone or in combination with invasive treatments such as surgery or embolization. Medical therapy thereby aims to reduce the proliferative potential especially in progressive stages of AVM. Although there is no evidence from larger prospective cohorts, there is an increasing amount of reports describing the successful medical treatment of AVMs. Targeting the PI3KCA (Alpelisib), mTOR (Sirolimus), MEK (Trametinib), BRAF (Dabrafenib) and VEGF (Bevacizumab) pathways are currently being explored as off-label treatments and patients scheduled for one of those treatments can subsequently be included in the study [21, 32–34].

Image-guided embolization is performed under general anesthesia following standard operating procedures at each center. Access to the lesion will either be gained via a transarterial, transvenous, or percutaneous route, or a combination. Embolization thereby always aims at complete closure of the nidus, and if possible, including closure of draining veins and feeding arteries. For this purpose, antegrade or retrograde plug-and push technique, pressure cooker technique as well as double lumen balloon catheters can be applied [28, 35, 36]. Application of embolization devices is restricted up to the dose provided by the manufacturer. Depending on the AVM extent, morphology, and flow characteristics, multiple treatment cycles may be required to achieve complete or near-complete occlusion of the nidus. Particularly in case of extensive lesions there may be no adequate clinical improvement after one initial embolization. Besides that, complete embolization should aim at minimizing the risk for recurrence in general. All agents used for embolization purposes are CE-marked. Surgery is similarly conducted under general anesthesia and carried out by corresponding specialties such as plastic surgery, otorhinolaryngology/head and neck surgery, maxillofacial surgery, orthopedics, pediatric and general surgery.

Currently, there is no evidence for best practice; however, the study group agreed upon the following principles for invasive AVM treatment:

- Only if surgery is expected to achieve a complete removal of the AVM, resection can be considered as the first-line treatment choice.
- In cases where surgical removal of the AVM is not considered feasible, embolization is considered the first line therapy.
- Embolization of the nidus is the primary goal; mere occlusion of feeding arteries shall be avoided. Embolization materials therefore have to be selected in order to achieve this goal, and ethylene vinyl alcohol copolymer (EVOH)-based agents are considered the most proper ones.
- As first studies suggest that embolization with subsequent resection of the embolized AVM may be beneficial and reduce recurrence rates, embolization and subsequent surgery will be aimed at when possible [26, 37].
- Reconstructive surgery shall be performed only after near complete embolization of the AVM.

Center specific differences in treatment will not be avoided, thus reflecting a real-world setting for the presented study.

### Follow-Up

**Fig. 1:**
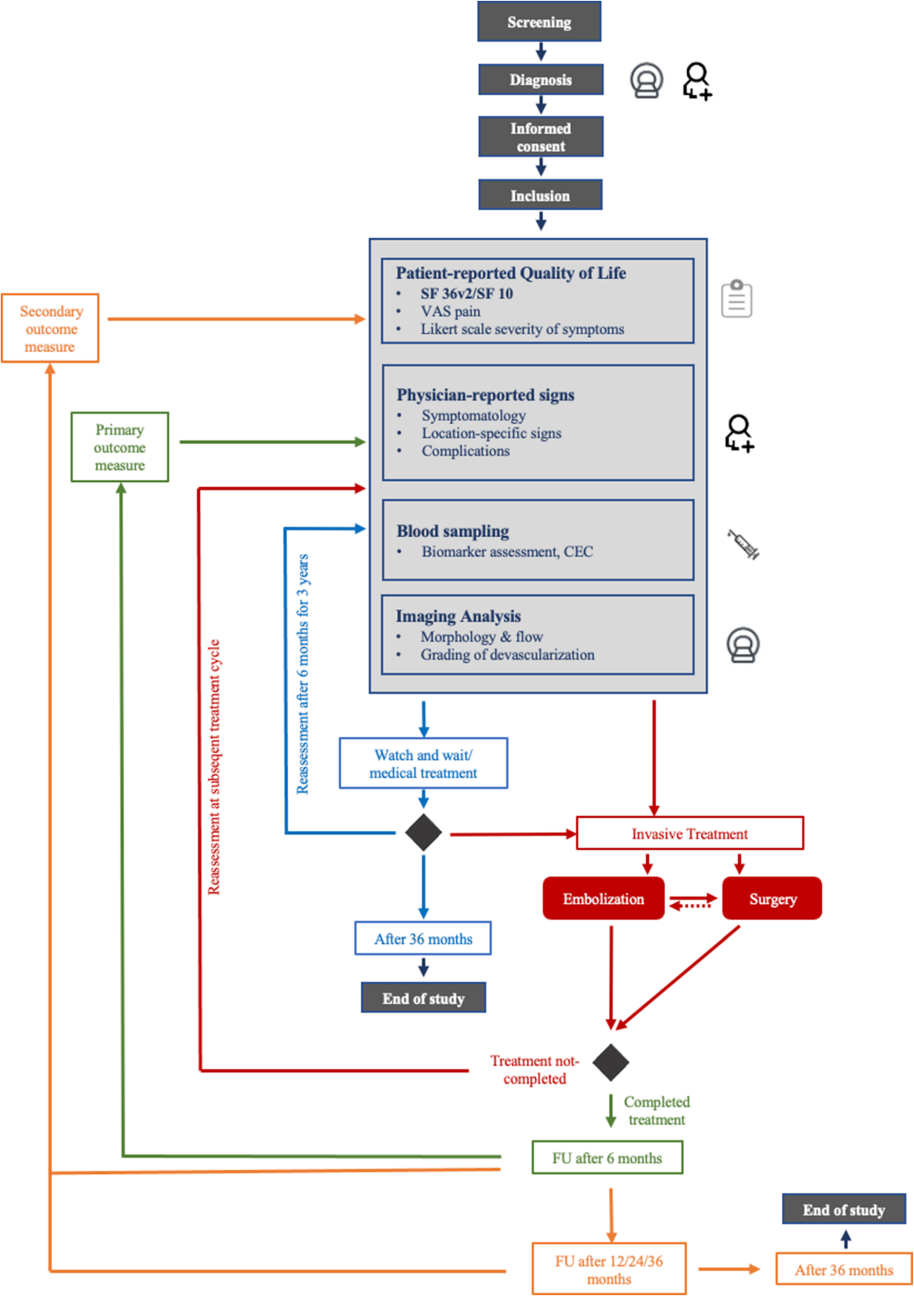
Schematic overview of multimodal treatment modalities and major time points for study participants. Quality of Life (patient-reported), clinical presentation (physician-reported signs), imaging (radiological assessment of devascularization), recurrence rate, and therapeutic safety (treatment-associated morbidity) will be analyzed at 6, 12, 24, and 36 months for both patients with watch and wait strategy (blue arrow) as well as patients undergoing invasive treatment (red arrow). As patients with invasive treatments receive additionally assessments at each invasive treatment cycle, those are presented separately in the figure. Of note, there are no definite treatment arms consisting of a defined treatment modality, but instead treatment modalities may be combined, e.g. a patient undergoing embolization first with subsequent surgical resection. Furthermore, at each time point, levels of obtained liquid biopsies for detection of potential serum biomarkers and circulating endothelial cells (CEC) will be analyzed

The follow-up will be performed following a dedicated timeline, see Fig. 1. In case of conservative management, all assessments will be conducted biannually for 36 months. If the patient receives interventional/surgical therapy, the assessments will take place prior to treatment initiation as well as before each treatment cycle. There are defined follow-ups every 6 months after initiation of interventional/surgical therapy. After completion of interventional/surgical therapy, follow-up is performed at 6 (primary outcome measure), 12, 24, and 36 months (secondary outcome measures). Data collection will be stopped in case of withdraw of informed consent.

## Outcome Measures

In order to investigate the QoL (primary outcome measure) in a standardized way, established questionnaires, the short form-36 version 2 (SF-36v2®) health survey (QualityMetric Inc., USA) for adults and children > 13 years as well as the SF-10 health survey for children™ (QualityMetric Inc, USA) aged 5–13 years, will be used.

The SF-36v2 health survey consists of eight scaled scores, which are the weighted sums of the questions in their section and yield two summary measures, the physical component summary (PCS) and the mental component summary (MCS).

The SF-10 is a 10-item questionnaire for children aged 5–13 years completed by caregivers. The scoring method yields two summary measures, the physical summary score (PHS) and the psychosocial summary score (PSS).

Secondary outcomes are clinical presentation (VAS scale of pain, physician-reported signs), AVM imaging (radiological assessment of devascularization), recurrence rate, and therapeutic safety (treatment-associated morbidity) as well as biomarker and CEC levels following therapy.

As physician-reported signs, the general appearance (swelling, pulsation, ulceration, cardiovascular health issues, bleeding) is assessed at each follow-up. Here, initial clinical manifestations and their further course (steady state, clinical improvement, progress) are documented within the case report forms (CRFs).

Imaging analysis consists of a detailed assessment of the therapeutic response. MR measurements will be performed to determine the reduction of AVM lesion size (volume) at all follow-up time points. AVM lesions will therefore be manually segment in transverse MR angiography images as well as post-contrast T1-weighted fat-saturated (fs) images (slice thickness 3–5 mm). Segmentation will be restricted to perfused areas, so that reduced perfusion will contribute to lesion size analysis. Manual segmentation with region of interest (ROI) placement in each plane will further allow semi-automated analysis of perfusion and enhancement. All aiming datasets will be collected in a pseudonymized way by the primary side and imaging data thus be analyzed by a central core lab, blinded to clinical and treatment data. MR imaging data will be analyzed by to independent board-certified radiologist in an independent manner. Besides the lesion size assessment as described above, the degree of AVM devascularization will be subjectively evaluated and compared from pre-treatment imaging to both primary and secondary outcome measures by two independent readers as follows: 100% devascularization, 76–99% devascularization, 50–75% devascularization, and < 50% devascularization.

It has to be acknowledged that currently radiological response assessment to AVM treatment is both not standardized and the few approaches being present in the literature are not validated. Thus, the APOLLON study additionally aims to collect a standardized set of imaging data which will later allow to evaluate various response assessment strategies and come up with a valid image-based assessment mode for AVMs.

The assessment of recurrence rates will include clinical symptoms and imaging response (size and perfusions characteristics). Similarly to radiologic imaging, there is no standardized definition of ‘recurrence’ in the field of AVM. For the study protocol recurrence has been defined either clinically, meaning if a patient reports recurrent progress in size, e.g., new pulsating lesions are being detected by the patient (or clearly documented by the treating physician), or if a progression in size is visible on MR angiography (or post-contrast T1-weighted fs images). Again, this progression has to be confirmed by two independent readers (in case of disagreement, consensus will the sought by including a third reader). Furthermore, recurrence rates depending on the kind of treatment, the type of embolic agent (in the case of interventional treatment), and the initial clinical presentation of the AVM (initial severity of symptoms) will be analyzed.

The assessment of the therapeutic safety includes treatment-associated morbidity such as temporary (vascular procedure complication requiring surgical management, local tissue necrosis, excessive swelling or pain, infection, bleeding, thrombosis, embolism due to unintended dislocation of the agent to the lung, other non-target embolization, and extrusion of embolization material through adjacent tissue) and permanent sequelae (motoric and sensory nerve injury, lasting joint movement restrictions, 'tattoo effect' [38]) as well as mortality. Additionally, short- (30 days) and long-term complications will be differentiated. Complications of endovascular treatments will be classified according to the CIRSE guidelines [39].

Peripheral venous blood samples for biomarker and CEC analysis (secondary outcomes) will be obtained as additional blood samples of 10 ml on the day before embolization as well as at all follow-up time points. Investigated biomarkers to be analyzed include angiopoietin-I, angiopoietin-II, S-endoglin, transforming growth factor beta (TGF-beta), integrin beta-3, ephrin-B2, hepatocyte growth factor (HGF), angiostatin, endostatin, endothelin, basic fibroblast growth factor (bFGF), vascular endothelial growth factor (VEGF), interleukin-6 (IL-6), platelet derived growth factor (PDGF), and PIK3CA including circulating free DNA. In general, the EDTA blood samples will be processed by centrifugation and subsequently frozen until subsequent biomarker analysis, while the separated blood samples for CEC assessment are processed using the semi-automated CellSearch™ system and their specially developed reagents (CELLSEARCH® Circulating Endothelial Cell Kit, Menari Silicon Biosystems S.p.A., Italy). The detection of CEC in the CellSearch™ system is based on immunodetection of different membrane-bound markers. In case genetic analysis of AVM tissue is be performed, the results will be considered in the analyses.

### Primary Outcome Measure

The primary outcome measure within the multimodal treatment of AVMs is the patient-reported health-related QoL at 6 months after completion of invasive treatment, see Fig. 1.

### Secondary Outcome Measures

The clinical presentation (physician-reported signs), AVM imaging (radiological assessment of devascularization), recurrence rate, and therapeutic safety (treatment-associated morbidity) will be analyzed at 6 months. Also at 12, 24, 36 months after completion of treatment, QoL and all the assessments above will be repeated and analyzed. Levels of obtained liquid biopsies for detection of potential biomarkers and CEC will be investigated.

## Statistics

Standard descriptive statistics will be used when reporting the study data. For continuous data, distribution parameters (mean, standard deviation, minimum, median, and maximum) will be computed and for categorical data, frequency counts will be given. If needed, 95% confidence intervals will be specified. For the primary study parameters (PCS and MCS scores from adult SF-36v2 as well as PHS and PSS scores from SF-10), paired t-tests will be calculated comparing the scores at baseline to the respective scores at 6 months after treatment completion (for embolized patients) or at the 24-month follow-up (for patients with conservative or medical treatment). All QoL scores and sub-scores, as well as patient and physician reported pain and symptom data will be tabulated by study visit. Where appropriate, such data will also be summarized for different patient subgroups (e.g., age group, Schobinger stage, study treatment, etc.). Morbidity, mortality, and recurrence rates will be compared for different patient subgroups using Chi-square or Fisher’s exact test. Summary statistics for AVM nidus volume reduction will be presented stratified by clinical outcome, as well as by patient subgroups, as needed. Cross-tabulations of AVM devascularization (100%/76–99%/50–75%/ < 50% devascularization) vs. different clinical outcomes will be presented. The evidence for certain vascular anomalies treatments is limited to a very small number of studies published. We used the studies by Bouwman et al. [25] and Wohlgemuth et al. [40] for sample size calculation. A sample size of *N* = 73 achieves 80% power to detect a mean of paired differences of 5 with an estimated standard deviation of differences of 15 and with a significance level (alpha) of 0.05 using a two-sided paired t-test. Due to the limited data being available currently, this calculation is preliminary and may be modified according to the results which are being achieved during the course of the study. All calculations are performed according to the standardized rules using QualityMetric Health Outcomes Scoring Software, version 4.5.1 (QualityMetric Inc., USA). All statistical testing will be performed based on a significance level of *α* = 5%.

## Discussion

The APOLLON trial was designed to collect prospective real-world data on the treatment of AVM, an orphan disease that affects patients for their entire lifetime and is associated with a high morbidity. The study protocol investigating established multimodal therapy used in clinical routine in a standardized manner offers the potential to provide improved understanding of the clinical outcome in this rare disease. This evidence may help to reduce morbidity and at the same time to increase the health-related QoL in patients with AVMs. Particularly, this applies with regard to the natural progress of the disease, which may lead to peripheral limb necrosis, tissue loss, arterial bleeding, and cardiac failure in untreated malformations. The prospective APOLLON study to assess the effectiveness, safety, and clinical outcome including health-related QoL is expected to generate evidence for minimally invasive, surgical, and conservative treatment concepts with the goal of improved patient selection as well as optimized patient care.

## Supplementary Information

Below is the link to the electronic supplementary material.

Supplementary file1 (DOCX 131 kb)

## Abbreviations

aPPT: Activated partial thromboplastin time
AVM: Arteriovenous malformation
bFGF: Basic fibroblast growth factor
CCI: Comprehension complication index
CD: Clavien–Dindo
CEC: Circulating endothelial cells
CRF: Case report form
ECOG: Eastern Cooperative Oncology Group
fs: Fat-saturated
FU: Follow-up
GFR: Glomerular filtration rate
HGF: Hepatocyte growth factor
HHT: Hereditary hemorrhagic telangiectasia
IL-6: Interleukin-6
INR: International normalized ratio
ISSVA: International Society for the Study of Vascular Anomalies
MCS: Mental component summary
MR(I): Magnetic resonance (imaging)
MAPK: Mitogen-activated protein kinase
PCS: Physical component summary
PDGF: Platelet derived growth factor
PHS: Physical summary score
PIK3CA: Phosphatidylinositol-4,5-bisphosphate 3-kinase catalytic subunit alpha
PSS: Psychosocial summary score
PTEN: Phosphatase and tensin homolog
QoL: Quality of life
RAS: Rat sarcoma
ROI: Region of interest
SF: Short form
TGF-beta: Transforming growth factor beta
VAS: Visual analogue scale
VEGF: Vascular endothelial growth factor
