# Supplementary material for: Clinical Outcome and Quality of Life of Multimodal Treatment of Extracranial Arteriovenous Malformations: The APOLLON Study Protocol
Source: Cardiovasc Intervent Radiol. 2022 Oct 19;46(1):142–51. doi: 10.1007/s00270-022-03296-8 (PMC9810564; doi:10.1007/s00270-022-03296-8)
Supplement: Supplementary file 1 — Supplementary file1 (DOCX 131 kb) [file 270_2022_3296_MOESM1_ESM.docx]

**Study Protocol**

**Apollon**

Clinical outcome and Quality of Life of multimodal treatment of arteriovenous malformations of the body

LMU University Hospital Munich

Klinik und Poliklinik für Radiologie

&

Abteilung für Hand-, Plastische und Ästhetische Chirurgie

Klinik und Poliklinik für Hals-Nasen-Ohrenheilkunde

Kinderchirurgische Klinik und Poliklinik im Dr. von Haunerschen Kinderspital

Head of clinical trial (Radiology): Prof. Dr. med. Moritz Wildgruber

Representative and coordinator (Radiology): Prof. Dr. med. Jens Ricke

Investigators (Radiology): Dr. med. Vanessa F. Schmidt

Prof. Dr. med. Max Seidensticker

Investigator (Hand-, Plastic-, Aesthetic Surgery): PD Dr. med. Denis Ehrl

Investigator (Otolaryngology/ENT Surgery): PD Dr. med. Frank Haubner

Investigators (Children's Hospital): Dr. med. Beate Häberle

Dr. med. Alexandra Pohl

Content

[1. Title 4](#_Toc36038817)

[1.1. Title of the research project 4](#_Toc36038818)

[1.2. German Clinical Trials Register 4](#_Toc36038819)

[2. Responsibilities 4](#_Toc36038820)

[2.1. Head of clinical trial 4](#_Toc36038821)

[2.2. Representative 4](#_Toc36038822)

[2.3. Internal Investigators 4](#_Toc36038823)

[2.4. External Investigators 5](#_Toc36038824)

[2.5. Participating departments 6](#_Toc36038825)

[3. Project rationale 8](#_Toc36038826)

[3.1. Background and state of the art 8](#_Toc36038827)

[3.2. Study Reason 11](#_Toc36038831)

[3.3. Risk-benefit-ratio 12](#_Toc36038832)

[4. Study objectives 13](#_Toc36038833)

[4.1. Primary study objectives 13](#_Toc36038834)

[4.2. Secondary study objectives 13](#_Toc36038835)

[4.3. Primary Endpoint: 14](#_Toc36038836)

[4.4. Secondary Endpoints 15](#_Toc36038837)

[5. Study design 16](#_Toc36038838)

[6. Study population 16](#_Toc36038839)

[6.1. Inclusion Criteria 16](#_Toc36038840)

[6.2. Exclusion Criteria: 17](#_Toc36038841)

[7. Study regulations 18](#_Toc36038842)

[7.1. Information and consent 18](#_Toc36038843)

[7.2. Description of the procedures 18](#_Toc36038844)

[8. Follow-up 18](#_Toc36038845)

[9. Adverse events (AE) / serious adverse events (SAE) 19](#_Toc36038846)

[9.1. Treatment in case of AE/SAE **Fehler! Textmarke nicht definiert.**](#_Toc36038847)

[9.2. Reporting process **Fehler! Textmarke nicht definiert.**](#_Toc36038848)

[10. Statistics 19](#_Toc36038849)

[11. Data management 20](#_Toc36038850)

[11.1. Data collection and storage 20](#_Toc36038851)

[11.2. Data transfer 21](#_Toc36038852)

[11.3. Archiving 21](#_Toc36038853)

[12. Ethical and legal aspects 22](#_Toc36038854)

[12.1. Declaration of Helsinki 22](#_Toc36038855)

[12.2. Qualification of the study site 22](#_Toc36038856)

[12.3. Termination criteria 23](#_Toc36038857)

[13. Data protection 23](#_Toc36038858)

[14. Insurance 23](#_Toc36038859)

[Literatur Fehler! Textmarke nicht definiert.](#_Toc36038860)

# Title

**Apollon**

## Title of the research project

Clinical outcome and Quality of Life of multimodal treatment of arteriovenous malformations of the body

## German Clinical Trials Register

DRKS00021019

# Responsibilities

## Head of clinical trial

**Prof. Dr. med. Moritz Wildgruber**

Klinik und Poliklinik für Radiologie

LMU Klinikum der Universität München

Marchioninistr. 15

D-81377 München

## Representative

**Prof. Dr. med. Jens Ricke**

Klinik und Poliklinik für Radiologie

LMU Klinikum der Universität München

Marchioninistr. 15

D-81377 München

## Internal Investigators

**Prof. Dr. med. Max Seidensticker**

**Dr. med. Vanessa F. Schmidt**

Klinik und Poliklinik für Radiologie

LMU Klinikum der Universität München

Marchioninistr. 15

D-81377 München

**PD Dr. med. Denis Ehrl**

Abteilung für Hand-, Plastische und Ästhetische Chirurgie

LMU Klinikum der Universität München

Marchioninistr. 15

D-81377 München

**PD Dr. med. Frank Haubner**

Klinik und Poliklinik für Hals-Nasen-Ohrenheilkunde

LMU Klinikum der Universität München

Marchioninistr. 15

D-81377 München

**Dr. med. Beate Häberle**

**Dr. med. Alexandra Pohl**

Kinderchirurgische Klinik und Poliklinik im Dr. von Haunerschen Kinderspital

LMU Klinikum der Universität München

Lindwurmstraße 4

D-80337 München

## External Investigators

Prof. Dr. Dr. Walter A. Wohlgemuth

Universitätsklinik und Poliklinik für Radiologie

Ernst-Grube-Straße 40

06120 Halle/Saale

Prof. Dr. René Müller-Wille

Institut für Diagnostische und Interventionelle Radiologie

Universitätsmedizin Göttingen

Robert-Koch-Straße 40

37075 Göttingen

Dr. Michael Köhler, Dr. Max Masthoff

Institut für Klinische Radiologie

Universitätsklinikum Münster

Albert-Schweitzer-Campus 1

48149 Münster

Prof. Dr. Maciej Pech

Universitätsklinik für Radiologie und Nuklearmedizin

Leipziger Straße 44

39120 Magdeburg

PD Dr. Wibke Uller

Institut für Röntgendiagnostik

Universitätsklinikum Regensburg

Franz-Josef-Strauss Alle11

93053 Regensburg

Prof. Dr. Maliha Sadick

Institut für Klinische Radiologie und Nuklearmedizin

Universitätsmedizin Mannheim

Theodor-Kutzer-Ufer

68167 Mannheim

Dr. Lars Maruschke/Dr. Friedrich Kapp

Klinik für Radiologie/Klinik für pädiatrische Hämatologie und Onkologie

Universitätsklinikum Freiburg

Hugstetter Straße 55

79106 Freiburg

## Participating Departments

Klinik und Poliklinik für Radiologie

LMU Klinikum der Universität München

Marchioninistr. 15

D-81377 München

Abteilung für Hand-, Plastische und Ästhetische Chirurgie

LMU Klinikum der Universität München

Marchioninistr. 15

D-81377 München

Klinik und Poliklinik für Hals-Nasen-Ohrenheilkunde

LMU Klinikum der Universität München

Marchioninistr. 15

D-81377 München

Kinderchirurgische Klinik und Poliklinik im Dr. von Haunerschen Kinderspital

LMU Klinikum der Universität München

Lindwurmstraße 4

D-80337 München

Universitätsklinik und Poliklinik für Radiologie
Ernst-Grube-Straße 40
06120 Halle/Saale

Institut für Diagnostische und Interventionelle Radiologie
Universitätsmedizin Göttingen
Robert-Koch-Straße 40
37075 Göttingen

Institut für Klinische Radiologie
Universitätsklinikum Münster
Albert-Schweitzer-Campus 1
48149 Münster

Universitätsklinik für Radiologie und Nuklearmedizin
Leipziger Straße 44
39120 Magdeburg

Institut für Röntgendiagnostik
Universitätsklinikum Regensburg
Franz-Josef-Strauss Alle11
93053 Regensburg

Institut für Klinische Radiologie und Nuklearmedizin
Universitätsmedizin Mannheim
Theodor-Kutzer-Ufer
68167 Mannheim

Klinik für Radiologie/Klinik für pädiatrische Hämatologie und Onkologie
Universitätsklinikum Freiburg
Hugstetter Straße 55
79106 Freiburg

# Project rationale

## Background and state of the art

Even in the presence of increasing awareness of rare or ‘orphan’ diseases, congenital vascular anomalies of the body remain a diagnostic and therapeutic challenge. Until now, no reliable data exists from national and international treatment centers, regarding the number of affected patients.

Congenital vascular anomalies are currently considered to be among the most complex vascular diseases. They include vascular tumors, based on endothelial cell proliferation, and vascular malformations with underlying mesenchymal and angiogenetic dysplasia^1,2^. Whereas vascular tumors may regress with patient´s age^3^, vascular malformations never regress on their own, but frequently increase in size^4^. They are subdivided in capillary, venous, lymphatic, arterio-venous and combined malformations, depending on the dominant vessel type^5^. According to their appearance, venous malformations are the most common representatives of vascular anomalies (70%), followed by lymphatic malformations (12%), arterio-venous malformations (8%), combined malformation syndromes (6%) and capillary malformations (4%)^5^. Vascular anomalies present with diverse symptoms and morphology which makes the correct clinical diagnosis challenging. The existing various terminologies and classification attempts have been hampering correct diagnostic and therapeutic management in the past^6^. A comprehensive and well-founded global classification system for congenital vascular anomalies is required to allow precise diagnosis and avoid inappropriate therapeutic decisions, especially in rare and multifaceted diseases involving various organ systems. In 2018, the International Society for the Study of Vascular Anomalies (ISSVA) last updated the existing classification and published a supplemented version with insights of vascular anomalies and causal genetic disorders of disease manifestation (https://www.issva.org/UserFiles/file/ISSVA-Classification-2018.pdf).

Vascular malformations, based on an angiogenetic and vasculogenetic dysplastic disorder, are always present at birth (even if asymptomatic) and never regress spontaneously. They may be quiescent for a long time, before mechanical or hormonal influence stimulates them to gain volume and size. With increasing size, vascular malformations can create pain and functional impairment which require therapy. Diagnosis has to address morphology, lesion extent, dominant vessel type and potential complications regarding dermal, orthopedic, neurological and organ manifestations of the lesion. Vascular malformations can be composed of a single vessel type, combined vascular components and malformations with additional non-vascular anomalies and are therefore classified as “simple” or “combined vascular malformations” as well as “vascular malformations associated with other anomalies”. Arterio-venous malformations (AVMs) are rare vascular anomalies, but undoubtedly, the most challenging to treat successfully. As they invariably progress due to the fast flow through the arteriovenous shunts, AVMs can cause various complications, ranging from local pain and discomfort to peripheral ischemia with tissue necrosis, arterial bleeding and in case of long-lasting AV shunting even cardiac failure due to pressure overload^7^. The Schobinger classification for AVMs is valuable for assessing the clinical condition of the vascular anomaly and potential indications to treat^8^. According to Schobinger, four stages are distinguished: stage I (clinical inactive AVM, local skin hyperthermia), stage II (increase of arterio-venous shunting, presence of pulsation and bruit), stage III (destructive AVM, manifestation of ulcerations, bleeding and pain), and stage IV (decompensated AVM, heart insufficiency or cardiac failure). AVMs are fast-flow vascular anomalies. With increasing size, it is increasingly difficult to circumscribe the area of the direct arteriovenous interfaces (“nidus”) of the AVM and to differentiate between feeding inflow arteries and draining outflow veins. Diagnostic imaging provides information on the location, extension, composition and diameter of the feeding and draining vessels to plan for the minimally invasive therapeutic approach to AVMs.

In certain cases AVMs may be associated with hereditary hemorrhagic telangiectasia (HHT), Parkes Weber syndrome (PWS) or Capillary Malformation – AVM syndrome.

AVMs are diagnosed primarily clinical and are subsequently confirmed by cross-sectional imaging such as ultrasound, computed tomography (CT) and Magnetic Resonance Imaging (MRI). Analysis of flow patterns is useful to establish the diagnosis of a fast-flow malformation^9^. Conventional intraarterial arteriography is generally performed immediately before interventional treatment. As arteriovenous malformations frequently penetrate various tissues and as recurrence rates after surgery are high, primary surgical resection has become less common. Nowadays the value of surgery lies in resection of previously embolized lesions that are not or only residually perfused, to manage complications following embolization as well as to reconstruct larger defects occurring after embolization^10^.

Once the decision for invasive treatment is made, complete closure of the AVM is the goal^11^. Incomplete embolization can result in a rapid increase of the lesion. First data in cerebral AVMs indicate that partial embolization results in a release of various angiogenic growth factors from the AVM which foster a progress of the lesion rather than regression^12-14^. The most important therapy approaches are summarized below.

### Conservative therapy: Compression garments can improve symptoms and quality of life. Management of chronic pain should be optimized by a pain specialist. At present, the use of mTOR inhibitors for the treatment of aggressive AVMs is less promising^15^. Novel biologicals targeting the effects of characteristic mutations in AVMs such as PIK3CA or RAS/MAPK may become pharmaceutical options in the future (Venot Q et al Nature 2018, Al-Olabi JCI 2018).

### Embolotherapy: Invasive therapy is indicated in patients with progressive symptoms according to the Schobinger classification. Historically, open surgical ligation and resection of the nidus had been considered the treatment of choice. However, embolization offers a less invasive way of treatment with lower morbidity and acceptable results. In some cases preoperative embolization is an option when complete surgical resection of the nidus is achievable thereafter.

The goal of endovascular embolotherapy is to occlude the nidus or fistulas completely. Commonly used agents are ethanol, N-butyl cyanoacrylate (NBCA) and ethylene-vinyl-alcohol-copolymer (EVOH). Additionally, coils or vascular plugs are needed in some cases, but those only occlude the feeding vessels and never reach the actual nidus, therefore the use is considered as adjuvant, mere coiling of AMVs is nowadays obsolete. Many authors use a combination of the following embolic agents for the endovascular treatment of complex fast-flow malformations.

- *Ethanol.* If injected in the right manner, ethanol is a very potent embolic agent for the occlusion of symptomatic fast-flow malformations. However, there is a high risk of tissue necrosis, nerve injury, and even cardiocirculatory arrest. Because of its low viscosity ethanol passes the nidus very quickly centrally into the lung circulation. Therefore, the pulmonary arterial pressure (PAP) should be monitored continuously during ethanol application. PAP above 25mmHg systolic can be found 10 to 15 minutes after application. To avoid side effects many interventionalists administer less than 0.5 ml per kg.
- *N-butyl cyanoacrylate (NBCA):* N-butyl cyanoacrylate (NBCA) and derivatives are liquid adhesive agents that polymerize irreversibly when exposed to blood. To adjust polymerization time and to enable fluoroscopic visibility NBCA is commonly mixed with lipiodol (ratio, 1:1 to 1:5). One major drawback of NBCA is the potential risk of catheter tip adhesion inside the lesion and the passive, flow-directed administration.
- EVOH Ethylene-vinyl-alcohol-copolymer (EVOH). EVOH is a non-adhesive liquid embolic agent mixed with dimethyl sulfoxid (DMSO) and radiopaque tantalum powder (Onyx, Squid). Compared to NBCA, EVOH has a longer casting time, allowing further penetration into the nidus. It can be administered slowly in a controlled fashion under fluoroscopy, ideally using road map techniques. Using the reflux of EVOH as a plug around the catheter tip, a retrograde flow of EVOH into the whole nidus against the blood-flow is possible ("plug and push technique.")^16^. However, EVOH has some disadvantages. The injection is painful and embolization is therefore performed under general anesthesia.
- *Plugs and coils:* Plugs and coils can be used in simple structured AVMs (type I), for example in pulmonary fast-flow malformations. They have also a role as an embolic agent for outflow occlusion (type II lesions).

*Embolization technique in general:* Most frequently access is gained via the transarterial route, transvenous access maybe additionally helpful in complex lesion. Direct puncture of the nidus is suitable in type II and IIIb lesions. Complete occlusion of the nidus should be achieved, but frequently requires multiple therapy sessions. Embolization of remote arteries should in general be avoided.

Surgery: As stated above surgery is predominantly attempted after successful complete or partial embolization^10^. Aim is to remove the bulk of the lesion, decrease the rate of recurrence after embolization as well as to manage complications. Localized lesions may be amenable for primary surgery if infiltration to adjacent tissue is limited. Plastic reconstructive means are especially important in lesions located in the head and neck area.

### Post procedural care: Postprocedural pain should be treated consequently. Close monitoring of the skin and neurovascular assessments are mandatory. After embolization and other invasive approaches surveillance imaging for recurrence is being performed long-term.

## Study Rationale

Owing to the rare incidence of arteriovenous malformations and additionally the heterogeneity of clinical presentations as well as existing therapeutic approaches no evidence currently exists for image-guided embolotherapy, which however is emerging as the primary treatment of choice. Currently no prospective studies exist investigating the technical and clinical outcome, complication rates and therapy-induced changes in health-related quality of life. Due to the limited evidence regarding the outcome of angiographically guided AVM treatment and at the same time the high morbidity of the disease, prospective evidence is urgently needed.

## Risk-benefit-ratio

Minimal-invasive treatment methods for arterio-venous malformations have significantly improved during the last years. The aim of AV malformations therapy is to reduce symptoms, prevent potential impairment of functional tissue and resulting complications and thus help patients to improve their quality of life. An incomplete, non-indexed or incorrect therapy often leads to a worsening of symptoms. A strict risk-benefit assessment is therefore necessary before any invasive therapy.

With advancements in the development of embolic agents and microcatheter techniques, embolization therapy has evolved as the mainstay of treatment for peripheral AVMs. Although less invasive and relatively safe, embolization carries the risk of displacement of embolic material with the risk of systemic and pulmonary embolism, occlusion of non-AVM vasculature with subsequent tissue injury, and systemic reaction to embolic agents. Bleeding and infections are rare but can evolve as severe complications. The risk of exposure to contrast material and ionizing ration is especially important in children and young adults.

Postembolization syndrome caused by tissue necrosis may be encountered after a successful interventional embolization. The symptoms of fever, leukocytosis, pain and nausea occur soon after embolization and typically resolve within a few days. Only close laboratory work-ups and clinical observation can differentiate postembolization syndrome from an infective complication.

The actual frequencies of the complications mentioned above is not known and the existing moderate knowledge is derived from retrospective patient cohorts with low sample size, normally from one center. Thus, there is an urgent need for data from larger cohorts, at best collected in prospective manner and from multiple experienced centers.

The present protocol of an established multimodal therapy concept used in clinical routine in interdisciplinary consensus offers the potential to at the same time reduce morbidity and increase individually the health-related quality of life in patients with AV malformations. In view of the natural progress of the disease, which untreated may lead to peripheral limb necrosis, tissue loss, arterial bleeding, and cardiac failure, the treatment benefits seem to outweigh the associated risks.

The prospective study to assess the clinical outcome, safety and health-related quality of life is expected to generate evidence for minimally-invasive treatment concepts with improved patient selection.

In addition, the multi-centre design of the study with the potential to assess a larger patient population will provide an improved understanding in the dynamics and natural course of this rare disease.

# Study objectives

The study objective is to determine the effectiveness, safety and clinical outcome including health-related Quality of Life of multimodal treatment of arteriovenous malformations. Therapy options include conservative management, medical treatment, minimally-invasive image-guided therapy (sclerotherapy, embolization) and surgery as well as combinations of all of the above. Of note, especially image-guided therapy routinely requires multiple therapy sessions. Clinical outcome shall therefore be assessed before initiation of treatment, during therapy (including in between multiple therapy sessions) as well as at defined time points after therapy of the AV malformation. Therapy assessment is based on the recommendations of the international core outcome set (OVAMA)^17^. Additionally, liquid biopsies are being obtained from peripheral blood to investigate potential biomarkers (cytokines, chemokines, growth factors) for therapy response or/and disease progression.

## Primary study objectives

To determine effectiveness and clinical outcome of AV malformation therapy we will evaluate the self-reported therapy success in form of the changes in overall *patient-reported* health-related Quality of life (QoL) as the primary study objective. In order to investigate the changes in QoL in a standardized way, psychometrically evaluated questionnaires will be used (SF-36v2: adults, SF-10: kids). The SF-36v2 and the SF-10 health survey are internationally approved generic questionnaires to assess subjective health.

## Secondary study objectives

To determine effectiveness and clinical outcome of multimodal treatment of vascular malformations the following points are additionally examined as secondary study objectives (based on OVAMA^17-20^):

- *Physician-reported* signs: general appearance (for example swelling, pulsation, ulceration, cardiovascular health issues, bleeding) and location-specific signs (for example extremity length discrepancy, physical impairment)
- Imaging Response: MR-angiographically measured reduction of the size (volume) of the nidus of the AVM and degree of devascularization, in case of superficial cutaneous lesions patient photographs will be considered additionally
- Concordance between clinical and imaging outcome
- Serum/Plasma levels and respectively change of levels of potential biomarkers for prediction of therapy response or/and disease progression in an exploratory manner
- Recurrence Rate following multimodal AVM treatment
- Other local and systemic side effects at the follow-up visit

Assessments of the points listed above will be conducted at the following time points:

- At time of first presentation to the vascular malformation clinic after inclusion in the study
- In case of conservative management biannually thereafter for 36 months
- Before initiation of interventional/surgical therapy as well as before each cycle of l treatment
- After initiation of interventional/surgical therapy every 6 months
- After completion of interventional/surgical therapy follow-up is performed at 6 (primary endpoint), 12, 24 and 36 months

To determine safety of multimodal treatment of vascular malformations the following points are additionally examined as secondary study objectives:

• Treatment-associated morbidity including temporary (e.g. vascular procedure complication requiring surgical management, local tissue necrosis, infection, bleeding, thrombosis, embolism due to unintended dislocation of the agent to the lung, cardiovascular systemic complications) and permanent sequalae (e. g. motoric and sensory nerve injury, lasting joint movement restrictions) and mortality

• Other acute local and systemic side effects peri- and postinterventional

## Primary Endpoint:

*Patient-reported* health-related Quality of Life in a multimodal therapy concept for AV malformation treatment at 6 months after completion of invasive treatment. In order to investigate the QoL in a standardized way, established questionnaires (SF-36v2, SF-10) will be used.

- Adults: SF-36 Version 2: The SF-36v2 for adults (> 13 years) consists of eight scaled scores, which are the weighted sums of the questions in their section. The eight sections are vitality (VT; feeling energetic, absence of fatigue), physical functioning (PF; extent to which health limits daily physical activities), bodily pain (BP; intensity of pain and effect on activities), general health perception (GH; current health status, health expectations and resistance to illness), physical role functioning (RF; extent to which physical health interfere with typical role functions), emotional role functioning (RE; extent to which emotional health interferes with typical role functions), social functioning (SF; extent to which health-related problems interfere with normal social activities) and mental health (MH; general mental health, including anxiety, depression, positive affects). These eight sections yield two summary measures:
  - physical component summary (PCS)
  - mental component summary (MCS).
- Kids: SF-10: The SF-10 is a 10-item questionnaire for children aged 5 – 13 years designed to measure health-related QoL in children. Unlike the SF-36v2 it should be completed by caregivers. The scoring method yields two summary measures:
  - physical summary score (PHS-10)
  - psychosocial summary score (PSS-10)

## Secondary Endpoints

- Therapeutic efficiency and clinical outcome of the selected therapeutic concept for AV malformation treatment at 12, 24, 36 months after completion of invasive treatment:
- *Physician-reported* signs: general appearance (for example swelling, pulsation, ulceration, cardiovascular health issues, bleeding) and location-specific signs (for example extremity length discrepancy, physical impairment)
- Radiological assessment
  - Evaluation of the therapeutic response depending on imagine outcome:
    - MR-angiographically measured reduction of the size (volume) of the nidus of the AVM
    - Degree of AVM devascularization (modified from Cho et al 2006 and 2008)^21,22^ before treatment to both primary and secondary endpoints:
      - 100% devascularization
      - 76-99% devascularization
      - 50-75% devascularization
      - < 50% devascularization
- Concordance between clinical and imaging outcome
- Changes in patient photographs (only qualitative analysis with Likert scale)
- Identification of potential biomarkers (as of now: Angiopoetin-I, Angiopoetin-II, S-endoglin, TGF-beta, Integrin beta-3, Ephrin-B2, HGF, Angiostatin, Endostatin, Endothelin, bFGF, VEGF, IL-6, PDGF, S100A8/9, S100B, PROX1, NRP2, PIK3CA including circulating free DNA) for prediction of therapy response or/and disease progression in an exploratory manner. For this purpose, an additional blood sample of about 10 ml blood is obtained for the study. In case of a clinically required biopsy of the vascular malformation, obtained material may similarly be assessed for additional tissue biomarkers.
- Recurrence rates:
  - Clinical recurrence (reappearance or aggravation of symptoms or findings which were initially regressive)
  - Imaging recurrence (recurrent increase in size or reperfusion of the vascular malformation after initial regression, occurrence of newly perfused AVM not detected before)
  - Recurrence rates/Progression after initial partial versus after complete response
  - Recurrence rates depending on the kind of treatment, the type of embolic agent (in the case of sclerotherapy, embolization), and the initial clinical findings of vascular malformation (initial size or the initial severity of symptoms)
- Other local and systemic side effects at the follow-up visit

Therapeutic safety of the selected therapeutic concept for AV malformation treatment

- Treatment-associated morbidity including temporary (vascular procedure complication requiring surgical management, local tissue necrosis, infection, bleeding, thrombosis, embolism due to unintended dislocation of the agent to the lung) and permanent sequalae (motoric and sensory nerve injury, lasting joint movement restrictions) and mortality
- Other acute local and systemic side effects

# Study design

The study is a **multicenter, exploratory, prospective open-label study**. Due to **the multimodal therapy concept**, a comparison of the different therapy approaches will be conducted.

# Study population

A total of 148 patients with AV malformations will be treated across centers, of which approximately 50 patients will be treated at LMU University Hospital Munich.

## Inclusion Criteria

- Age: > 4 years, < 70 years
- Patients with simple peripheral (=extracranial) arteriovenous malformations according to the ISSVA (International Society for the Study of Vascular Anomalies) classification. Each patient and the corresponding diagnosis, is discussed in an interdisciplinary board for vascular anomalies before initiation of treatment.
- Patients with combined vascular malformations and arteriovenous malformations associated with other anomalies (e. g. Parkes Weber, PTEN hamartoma, HHT) according to the ISSVA (International Society for the Study of Vascular Anomalies) classification
- Patients with first line therapy or patients with previous alternative therapies in whom the previous treatments did not lead to durable symptom improvement
  - Previous surgery or embolization are not an exclusion criterion, but these patients will only be included in case of a therapy-free interval of 12 months
- Adults and children will be similarly included

## Exclusion Criteria:

- Patients with AV malformations located in the central nervous system
- Patients with AV malformations located in abdominal parenchymal organs or the gastrointestinal tract
- Patients with other high-flow vascular anomalies (e. g. vascular tumors)
- Patients who have been previously treated by surgery or sclerotherapy/embolization within the last 12 months
- Patients with concomitant life-limiting diseases (such as cancer)
- Patients with acute inflammatory diseases or acute bacterial superinfection of an AVM related ulceration
- Patients with contraindications for invasive treatments
  - Patients with contrast agent intolerance or renal insufficiency (GFR>30ml/min)
  - Patients with impaired coagulation status:
    - Platelet count <50.000 / μl
    - aPTT > 50s
    - INR > 1,5
  - Poor general condition with ECOC performance > 1
- Pregnant or breast-feeding women
- Inability to access the AVM lesion due to anatomical or pathoanatomical reason
- Inability of the patient/parents to understand or follow the study protocol e.g. due to impaired mental health status
- Patients/parents who refuse to give informed consent

# Study regulations

## Information and consent

Patients are recruited according to the inclusion criteria and informed consent is obtained at least 24 hours before inclusion in the study. Age-appropriate consent forms are available. In case of age <18 years, parents and child are consented in accordance.

## Description of the procedures

Potential study participants are screened on an interdisciplinary basis according to the inclusion criteria through the participating centers. The screening includes laboratory analysis and pre-interventional imaging with US/CT/MRI to confirm the diagnosis and to identify possible treatment options, which are performed with the routine clinical workup. Furthermore, the exact size assessment of the initial extent of the vascular malformation (volumetric measurements, flow characteristics) is being performed based on the cross-sectional imaging data.

After detailed study information (orally and written) and informed consent, the patient is included in the study. The pre-therapeutic assessment is composed of the same subjective (SF-36v2/SF-10, VAS, Likert scale of severity of symptoms) and objective (general appearance and location-specific signs) surveys that are done during the follow-up appointments. Following interdisciplinary consensus, multimodal treatment is initiated

**Compression treatment** is considered as the baseline therapy for all vascular malformations. Patients are supplied with custom made compression garments (class II). Depending on severity of disease, permanent compression therapy may be applied or temporary compression may be considered sufficient.

**Embolization** is performed under general anesthesia following standard operating procedures. Access to the lesion will either be gained via a transarterial, transvenous or percutaneous route, or a combination of the latter. For transarterial or transvenous access is gained via a femoral or brachial access using a 5-8 French-sized catheter equipment. If additional percutaneous access is required lesion will be punctured under ultrasound guidance using a 20-21G needle. After contrast injection digital subtraction angiograms are acquired and subsequently embolization is performed under fluoroscopy guidance. Periprocedural anticoagulation is performed using unfractionated heparin in a weight-adapted manner. Periprocedural antibiosis is not applied routinely. Depending on the extent and morphology of the lesion multiple treatment sessions will be required in order to achieve complete/near-complete embolization. All agents used for embolization purposes are CE-marked.

In case of **surgical resections** of AVM tissue, surgery is performed after complete/near-complete embolization. Surgical resection is similarly performed under general anesthesia. Neuromonitoring will be routinely used in case of proximity of the AVM to major nerve structures. Surgical resection is limited to mass-forming AVM tissue, complete resection of the entire malformation is rarely possible and not goal of the procedure.

Peripheral venous blood samples will be obtained on the day before, on day 1 and 3 following embolization as well during follow up at the same time points of health related QoL assessment. Blood samples will be processes and subsequently frozen until subsequent biomarker analysis.

# Follow-up

Follow-up will be performed following a dedicated time line:

- In case of conservative therapy biannually for 36 months
- Before initiation of interventional therapy as well as before each cycle of interventional treatment
- After initiation of interventional therapy every 6 months
- After completion of interventional therapy follow-up is performed at 6 (primary endpoint), 12 , 24 and 36 months

A flowchart with the corresponding clinical, laboratory and radiological examinations is attached at page 24.

# Adverse events (AE) / serious adverse events (SAE)

Adverse events are considered to be directly or indirectly related to the study protocol. A serious adverse event is considered to be a study-related AE that:

· leads to death.

· is life-threatening (the danger to life must have actually existed; it is not enough that the event could have led to death if it would have been more serious).

· requires hospitalization or prolongs a hospital stay.

· leads to a permanent and significant disability.

The invasive therapy regimens as listed above can cause a variety of side effects and procedure related complications. As those events are however not related to the observational character of the study protocol, those are not considered to be Adverse Events (AE) or Serious Adverse Events (SAE).

Procedure-related events, not considered to be AE/SAE:

· Bleeding up to hypovolemic shock with indication for blood transfusion or emergency intervention/surgery

· Infections with consecutive systemic spread up to sepsis/shock including septic arthritis

· Damage caused by ionizing radiation (especially radiation dermatitis)

· Unforeseen Injury to tissue adjacent to the AVM, especially soft tissue, skin, vessels and nerves

· Unforeseen cardiovascular disorders such as pulmonary embolism and stroke

· Contrast medium or drug-induced renal failure, which may require dialysis treatment

· Contrast medium-induced thyroid disorders

· Allergic reactions

# Statistics

Analysis of clinical patient data, QoL data and Biomarkeranalysis will be performed separated from each other in a blinded setting. The study has an exploratory focus. All statistical testing will be performed based on a significance level of α=5%. No adjustment for multiple testing is planned.

Standard descriptive statistics will be used when reporting the study data. For continuous data, distribution parameters (mean, standard deviation, minimum, median, and maximum) will be computed and for categorical data, frequency counts will be given. If needed, 95% confidence intervals will be specified.

For the primary study parameters (scores PCS and MCS from adult SF-36v2 quality of life questionnaires, as well as PHS and PSS from kids SF-10 questionnaires), paired t-tests will be calculated comparing the scores at baseline to the respective scores at 6 months after the end of treatment (for embolized patients) or at the 24 months FU (for patients with conservative or medical treatment).

All QoL scores and subscores, as well as patient and physician reported pain and symptom data will be tabulated by study visit. Where appropriate, such data will also be summarized for different patient subgroups (e.g., age group, Schobinger stage, study treatment, etc.)

Morbidity, Mortality, and recurrence rates will be compared for different patient subgroups using Chi Square or Fisher’s exact test.

Summary statistics for AVM nidus volume reduction will be presented stratified by clinical outcome, as well as by patient subgroups, as needed. Crosstabulations of AVM devascularization (100% / 76-99% /50-75% / < 50% devascularization) vs. different clinical outcomes will be presented.

A sample size of N=74 (for children and adults, respectively) achieves 80% power to detect a mean of paired differences of 5 with an estimated standard deviation of differences of 15 and with a significance level (alpha) of 0,05 using a two-sided paired t-test.

# Data management

In accordance with the German Federal Data Protection Act (BDSG), there are taken all necessary measures to protect the individual from the fact that his or her personal rights are impaired by the handling of his or her personal data. The personal data are recorded pseudonymously.

## Data collection and storage

The collected findings, measurement results and all data collected according to the study plan are entered into the Case Report Forms (CRFs). The number and sequence of the planned examinations can be taken from the examination design and correspond to the routine procedure. Because of the data volume and the planned time frame of the study it is necessary to support both the study monitoring and the biometric evaluation with a database. This is intended to ensure the timely collection and evaluation of study data and to guarantee continuous monitoring for data completeness and accuracy. With the database it is also possible to do some exploratory data analysis from the beginning of the study.

## Data transfer

Patients sign a declaration of informed consent in which they agree to participate in the study. Furthermore, after detailed explanation, the patients agree that their data may be used for evaluation and subsequent publication in a pseudonymized form. If such consent is not given by the patient, no study-related data will be stored or evaluated. In addition, no data is passed on to external cooperation partners.

## Data scoring

The basic scoring algorithm is according to the numerical coding of responses to survey items (questions) and the formulas for combining item scores to produce scale scores (Saris-Baglama 2011). For the SF-36v2, eight profile scales and two component summary measures (PCS and MCS) are produced (Ware 2000) whereas two summary measures (PHS and PSS) are calculated for the SF-10 (Landgraf 1996).

Scoring is standardized in the SF-36v2 using the means and standard deviations from the 2009 U.S. general population surveys. The T-score of 50 corresponds to the average score of the U.S. general population with a standard deviation of 10. T-score based scoring is also used to score the SF-10 Health Survey for Children summary scales (Saris-Baglama 2011). The scale scores have been centered: a score of 50 corresponds to the average score in a comprehensive sample in children.

Item internal consistency is established when the correlation between an item and its hypothesized scale is greater or equal to .40. This data quality indicator is considered satisfactory when at least 90 % of the hypothesized item-scale correlations are .40 or greater.

Tests of item discriminant validity were performed to test the validity of the hypothesized item groupings. This data quality indicator is considered satisfactory when at least 80 % of the hypothesized item-scale correlations are higher than the alternative item-scale correlations.

Cronbach’s alpha coefficient is an estimate of internal consistency reliability based on the number of items in a scale and the item homogeneity (similarity). A Cronbach’s alpha coefficient of greater or equal to .70 establishes scale reliability. The calculation of Cronbach’s alpha is affected by the number of items in a scale and the degree to which the items contained in the scale measure the same construct (item homogeneity). Thus, for each of the eight SF-36v2 profile scales a table is calculated with the number of items in the scale, the internal consistency reliability coefficient (Cronbach’s alpha) and the average inter-item correlation for the scale. These calculations are not performed in the SF-10 for children as subscales are missing in this questionnaire.

All calculations are performed according to the standardized rules using QualityMetric Health Outcomes Scoring Software, Version 4.5.1 (QualityMetric Inc., Lincoln, RI, USA). A paired two-sided student t-test of the transformed scales is applied to compare the results before and after treatment. A p-value < 0.05 was considered significant.

## Archiving

The pseudonymised data are stored in a study database.

# Ethical and legal aspects

## Declaration of Helsinki

In the currently valid version the principles of the Declaration of Helsinki are followed.

## Ethical considerations

Before patients are included in this study themselves and / or their parents / guardians are personally informed about the study and all necessary steps including follow-up with a standardized form. If they agree to attend, the written informed consent is documented. If they decline to attend to the study, neither the patients nor their parents / guardians have any disadvantages in their respective diagnostic workup or therapy. Participants of the study are not privileged as compared to others. Includes patients and / or their parents /guardians are informed that the additional collected data is of scientific interest only without direct clinical impact for their therapy. Data will be collected only when the condition of the patient is appropriate and does not lead to any delay in further diagnostic or therapeutic workup.

## Qualification of the study site

The multimodal treatment and evaluation is being performed by experienced clinicians and investigators.

The head of clinical trial has extensive experience in conducting clinical trials. The collected findings, measurement results and all data collected according to the study plan are entered into the Case Report Forms (CRFs). The number and sequence of the planned study visits can be taken from the examination design and correspond to the routine procedure.

## Termination criteria

Serious and unexpected events in the patient population are subject to a thorough examination of their causality and could lead to the discontinuation or termination of the study. The criteria correspond to GCP or the medicinal products act.

# Data protection

This study complies with the regulations on medical confidentiality and data protection. Personal data and findings are pseudonymized after the survey and only processed in this form. Access to the original data and the encryption key is limited to the contact persons mentioned above. The documents are kept in the central investigator folder for the duration of the study and they are destroyed after the legal retention period has expired. Decoding takes only place in cases where it is necessary because of the safety of the test person („medical reasons") or if there are changes in the scientific question („scientific reasons"). In the individual case it depends on the decision of the head of clinical trial (Prof. Dr. Wildgruber) whether circumstances exist which make it necessary to unblind study data. In case of publication of the study results, the confidentiality of personal data remains guaranteed.

# Insurance

A patient insurance is not intended for this study.

**Financial disclosure statement**

The signatories declare that they have no financial or economic interests with the manufacturers of the products used in the planned study.

Munich, 4th May 2020

| **Prof. Dr. med. Moritz Wildgruber** | Stamp and signature |
| --- | --- |
| **Prof. Dr. med. Jens Ricke** | Stamp and signature |
| **Prof. Dr. med. Max Seidensticker** | Stamp and signature |
| **Dr. med. Vanessa Franziska Schmidt** | Stamp and signature |
| **PD Dr. med. Denis Ehrl** | Stamp and signature |
| **PD Dr. med. Frank Haubner** | Stamp and signature |
| **Dr. med. Beate Häberle** | Stamp and signature |
| **Dr. med. Alexandra Pohl** | Stamp and signature |

I certify that the information provided in this application is correct. I believe that it is possible to conduct the above study in accordance with the protocol, national legislation and the principles of good clinical practice.

**Prof. Dr. med. Moritz Wildgruber**

Department of Radiology

LMU University Hospital Munich

Marchioninistr. 15

D-81377 Munich

**Legend (Flowchart 1 and 2)**

[1] Severity of symptoms: subjective likert scale ranging from 0 to 5; 0 = no symptoms, 1 = very mild symptoms, 2 = mild symptoms, 3 = moderate symptoms, 4 = severe symptoms, 5 = very severe symptoms

[2] patient-reported pain: visual analogue scale (VAS)

[3] General routine: small blood count, Coagulation, Electrolytes, CRP, TSH

[4] Specific blood assessment (biomarker): Angiopoetin-I, Angiopoetin-II, S-endoglin, TGF-beta, Integrin beta-3, Ephrin-B2, HGF, Angiostatin, Endostatin, bFGF, VEGF, IL-6, PDGF, S100B, PROX1, NRP2, PIK3CA protein

[5] Evaluation of the imagine outcome: MR-angiographically measured reduction of the size (volume) of the nidus of the AVM and the degree of AVM devascularization (modified from Cho et al 2006 and 2008) before treatment to both primary and secondary endpoints: 100% devascularization, 76-99% devascularization, 50-75% devascularization, < 50% devascularization

[6] the treatment procedure is not part of the observational study

[7] - if further intervention is indicated

- possible to skip if a further intervention is carried out within 30 days before/after the 6-month deadline

[8] - if further intervention is indicated

Literature:

1 Greene, A. K., Liu, A. S., Mulliken, J. B., Chalache, K. & Fishman, S. J. Vascular anomalies in 5,621 patients: guidelines for referral. *Journal of pediatric surgery* **46**, 1784-1789, doi:10.1016/j.jpedsurg.2011.05.006 (2011).

2 Ricci, K. W. Advances in the Medical Management of Vascular Anomalies. *Seminars in interventional radiology* **34**, 239-249, doi:10.1055/s-0037-1604297 (2017).

3 Wildgruber, M., Sadick, M., Muller-Wille, R. & Wohlgemuth, W. A. Vascular tumors in infants and adolescents. *Insights into imaging* **10**, 30, doi:10.1186/s13244-019-0718-6 (2019).

4 Johnson, C. M. & Navarro, O. M. Clinical and sonographic features of pediatric soft-tissue vascular anomalies part 1: classification, sonographic approach and vascular tumors. *Pediatric radiology* **47**, 1184-1195, doi:10.1007/s00247-017-3885-y (2017).

5 Adams, D. M. *et al.* Vascular anomaly cases for the pediatric hematologist oncologists-An interdisciplinary review. *Pediatric blood & cancer* **65**, doi:10.1002/pbc.26716 (2018).

6 Nosher, J. L., Murillo, P. G., Liszewski, M., Gendel, V. & Gribbin, C. E. Vascular anomalies: A pictorial review of nomenclature, diagnosis and treatment. *World journal of radiology* **6**, 677-692, doi:10.4329/wjr.v6.i9.677 (2014).

7 Weitz, N. A. *et al.* Clinical spectrum of capillary malformation-arteriovenous malformation syndrome presenting to a pediatric dermatology practice: a retrospective study. *Pediatric dermatology* **32**, 76-84, doi:10.1111/pde.12384 (2015).

8 Gilbert, P., Dubois, J., Giroux, M. F. & Soulez, G. New Treatment Approaches to Arteriovenous Malformations. *Seminars in interventional radiology* **34**, 258-271, doi:10.1055/s-0037-1604299 (2017).

9 Sadick, M., Muller-Wille, R., Wildgruber, M. & Wohlgemuth, W. A. Vascular Anomalies (Part I): Classification and Diagnostics of Vascular Anomalies. *RoFo : Fortschritte auf dem Gebiete der Rontgenstrahlen und der Nuklearmedizin* **190**, 825-835, doi:10.1055/a-0620-8925 (2018).

10 Goldenberg, D. C. *et al.* Surgical treatment of extracranial arteriovenous malformations after multiple embolizations: outcomes in a series of 31 patients. *Plastic and reconstructive surgery* **135**, 543-552, doi:10.1097/prs.0000000000000890 (2015).

11 Muller-Wille, R., Wildgruber, M., Sadick, M. & Wohlgemuth, W. A. Vascular Anomalies (Part II): Interventional Therapy of Peripheral Vascular Malformations. *RoFo : Fortschritte auf dem Gebiete der Rontgenstrahlen und der Nuklearmedizin*, doi:10.1055/s-0044-101266 (2018).

12 Kim, G. H. *et al.* Plasma levels of vascular endothelial growth factor after treatment for cerebral arteriovenous malformations. *Stroke* **39**, 2274-2279, doi:10.1161/strokeaha.107.512442 (2008).

13 Liu, A. S., Mulliken, J. B., Zurakowski, D., Fishman, S. J. & Greene, A. K. Extracranial arteriovenous malformations: natural progression and recurrence after treatment. *Plastic and reconstructive surgery* **125**, 1185-1194, doi:10.1097/PRS.0b013e3181d18070 (2010).

14 Sandalcioglu, I. E. *et al.* Proliferation activity is significantly elevated in partially embolized cerebral arteriovenous malformations. *Cerebrovascular diseases (Basel, Switzerland)* **30**, 396-401, doi:10.1159/000319568 (2010).

15 Gabeff, R. *et al.* Efficacy and Tolerance of Sirolimus (Rapamycin) for Extracranial Arteriovenous Malformations in Children and Adults. *Acta dermato-venereologica* **99**, 1105-1109, doi:10.2340/00015555-3273 (2019).

16 Wohlgemuth, W. A. *et al.* The retrograde transvenous push-through method: a novel treatment of peripheral arteriovenous malformations with dominant venous outflow. *Cardiovascular and interventional radiology* **38**, 623-631, doi:10.1007/s00270-015-1063-x (2015).

17 Horbach, S. E. R. *et al.* Development of an international core outcome set for peripheral vascular malformations: the OVAMA project. *The British journal of dermatology* **178**, 473-481, doi:10.1111/bjd.16029 (2018).

18 Horbach, S. E. R. *et al.* Outcome measurement instruments for peripheral vascular malformations and an assessment of the measurement properties: a systematic review. *Quality of life research : an international journal of quality of life aspects of treatment, care and rehabilitation* **29**, 1-17, doi:10.1007/s11136-019-02301-x (2020).

19 Lokhorst, M. M., Horbach, S. E. R., van der Horst, C. & Spuls, P. I. Finalizing the international core domain set for peripheral vascular malformations: the OVAMA project. *The British journal of dermatology* **181**, 1076-1078, doi:10.1111/bjd.18043 (2019).

20 Lokhorst, M. M. *et al.* Responsiveness of quality-of-life measures in patients with peripheral vascular malformations: the OVAMA project. *The British journal of dermatology*, doi:10.1111/bjd.18619 (2019).

21 Cho, S. K. *et al.* Peripheral arteriovenous malformations with a dominant outflow vein: results of ethanol embolization. *Korean journal of radiology* **9**, 258-267, doi:10.3348/kjr.2008.9.3.258 (2008).

22 Cho, S. K. *et al.* Arteriovenous malformations of the body and extremities: analysis of therapeutic outcomes and approaches according to a modified angiographic classification. *Journal of endovascular therapy : an official journal of the International Society of Endovascular Specialists* **13**, 527-538, doi:10.1583/05-1769.1 (2006).
